# Increased risk of acute myocardial infarction in Swedish patients with systemic sclerosis: a population-based study

**DOI:** 10.1093/rap/rkaf054

**Published:** 2025-05-09

**Authors:** Majd Bairkdar, Karina Patasova, Pontus Andell, Marie Holmqvist

**Affiliations:** Clinical Epidemiology Division, Department of Medicine, Solna, Karolinska Institutet, Stockholm, Sweden; Clinical Epidemiology Division, Department of Medicine, Solna, Karolinska Institutet, Stockholm, Sweden; Department of Physiology and Pharmacology, Karolinska Institutet, Stockholm, Sweden; Medical Unit Cardiology, Karolinska University Hospital, Stockholm, Sweden; Clinical Epidemiology Division, Department of Medicine, Solna, Karolinska Institutet, Stockholm, Sweden; Medical Unit Gastroenterology, Dermatology and Rheumatology, Karolinska University Hospital, Stockholm, Sweden

**Keywords:** SSc, acute myocardial infarction, ischaemic heart disease, cohort study

## Abstract

**Objectives:**

To study the risk of acute myocardial infarction (AMI) in patients with SSc in a population-based cohort.

**Methods:**

Using nationwide Swedish registers, we identified patients with incident SSc 2004–19 and age- and sex-matched comparators from the general population (1:10). Our primary outcome was incident AMI or death from incident AMI. We started follow-up from SSc diagnosis until the primary outcome, death from other cause than AMI, emigration or study end (31 December 2019). We estimated crude AMI incidence rate. We used flexible parametric models to explore the relative risk of AMI over time since diagnosis. We also used age as time scale to explore how AMI risk changes over increasing age. We also studied the outcomes of AMI in SSc compared with the matched comparators.

**Results:**

We identified 1579 patients and 16 064 comparators. The incidence rate of AMI was 75.2 (95% CI 58.8–94.6) per 10 000 person-years in patients with SSc and 37.5 (95% CI 34.0–41.3) in the comparators, median follow-up was 5.2 and 6.3 years, respectively. The adjusted hazard ratio (HR) was highest during the first year after SSc diagnosis (HR 3.1, 95% CI 2.0–4.6). In patients with SSc, the risk of AMI increased more rapidly with increasing age compared with the comparators. AMI in SSc was associated with higher risk of mortality (HR 2.7, 95% CI 1.6–4.4) but not 30-day readmission (HR 1.3, 95% CI 0.7–2.0) compared with the comparators.

**Conclusion:**

In line with previous studies, SSc is associated with a 2-fold increase in AMI incidence compared with the general population.

Key messages

- SSc is associated with increased risk of AMI compared to the general population.
- AMI risk increases more rapidly over increasing age in SSc compared to the general population.
- This study also confirms that AMI could be an early manifestation to consider in SSc.

## Introduction

SSc is a rare autoimmune rheumatic disease with a heterogeneous clinical presentation and pathophysiology characterized by fibrosis and microvascular changes [1]. SSc is associated with one of the highest mortality rates among the rheumatic diseases [2], and SSc-related cardiac involvement is one of the leading causes of death in this patient population [3]. Cardiac involvement has also been reported as a poor prognostic factor [4]. Several cardiac pathologies have been reported; myocardial inflammation, myocardial fibrosis and microvascular changes in addition to perfusion and reperfusion injury [5].

SSc is associated with a higher risk for acute myocardial infarction (AMI) compared with the general population independent of traditional risk factors for AMI [6–9]. These findings suggest that SSc is independently implicated in the increased risk for AMI. While prior studies often report the relative risk, there is limited information about the absolute risk of incident AMI and its progression over time since SSc diagnosis. These data are crucial for both rheumatologists and patients with SSc to better understand and assess individual patient risk. It remains unclear how the risk of AMI develops over increasing age in patients with SSc compared with the general population. Therefore, we aimed to study the risk of developing AMI in a population-based cohort of patients with SSc without a prior history of ischaemic heart disease compared with the general population in Sweden. We also aimed to study the clinical outcomes of AMI in patients with SSc compared with the general population.

## Methods

### Setting and study design

All residents in Sweden have equal access to the publicly funded healthcare system and are assigned a unique personal identity number allowing for linkage of different registers. This study is a population-based cohort study using data from several nationwide Swedish registers. This study was approved by the Swedish Ethical Review Authority, number 2020–04529. Patient consent was not required.

### Data sources

The National Patient Register (NPR) includes complete data on all hospitalizations in Sweden with complete coverage since 1997 in addition to outpatient specialized care visits with almost complete coverage since 2001. For every hospitalization or visit, a main diagnosis and up to 30 secondary diagnoses are registered according to the International Statistical Classification of Diseases and Related Health Problems (ICD) codes [10]. The Cause of Death Register (CDR) collects data on deaths using death certificates, with almost complete coverage since 1952. In the CDR, both the underlying cause of death and the contributing causes are recorded according to ICD codes [11]. The 10th revision of the ICD was introduced in Sweden in 1997. The Total Population Register (TPR) contains demographic information including birth year, sex and dates of migration [12]. The Longitudinal Integrated Database for Health Insurance and Labour Market Studies (LISA) collects annually data on education for all adults registered in Sweden ≥ 16 years since 1990 with high accuracy of highest attained education level [13]. The National Prescribed Drug Register (NPDR) provides data on all dispensed prescriptions in Sweden since 1 July 2005, recorded according to ATC codes (Anatomical Therapeutic Chemical Classification System) in addition to date of prescription, date of dispensation, package, etc. [14].

### Participants

We used the NPR to identify all patients with incident SSc in Sweden between 2004 and 2019. To be classified as incident SSc, we required at least two visits/hospitalizations with SSc coded as the main diagnosis (ICD-10: M34.0, M34.1, M34.8, M34.9), the first ever visit between 1 January 2004 and 31 December 2019 and the second visit within 1 year of the first. We required at least one of the two visits to be made in a rheumatology or internal medicine clinic. We included only individuals ≥ 18 years at the date of the second visit. We used the TPR to identify 1:10 comparators from the general population matched on birth year and sex. To ensure only individuals with no history of ischaemic heart disease are included, we excluded patients with SSc and comparators who had any visit indicating ischaemic heart disease as main or secondary diagnosis (ICD-10: I20-I25) prior to the index date (see below for definition).

### Exposure, start of follow-up and outcome

The exposure was incident diagnosis of SSc as defined above. Index date, start of follow-up, was the date of the second visit indicating SSc, and the respective comparators were assigned the same date. The primary outcome was incident AMI. The validity of AMI diagnosis using register data has previously been reported to be high, with a positive predictive value over 95% and a sensitivity of 77–92% using varying definitions, some of which also used the CDR [15]. We defined AMI as either a hospitalization with a discharge code indicating AMI as either the main or secondary diagnosis or a record in the CDR where AMI was coded as the underlying or the contributing cause of death (ICD-10: I21-I22). We followed all participants from the index date until the primary outcome or until being censored due to death from other cause than AMI, emigration or last date of follow-up (31 December 2019), whichever came first.

### Other covariates

We collected information on age at index date (as a continuous variable), sex (women *vs*. men) and education level in the year of index where we categorized the highest attained education variable into ≤ 9 years, 10–12 years and >12 years. We identified the following comorbidities at index date, known as risk factors for AMI: diabetes mellitus, hypertension, dyslipidaemia and renal disease, from the NPR and the NPDR. The definitions are provided in detail in Supplementary Data S1, available at *Rheumatology Advances in Practice* online. Since data on prescription from the NPDR are available since 1 July 2005, analyses comprising those covariates were limited to participants with index date starting from 1 January 2006, allowing for a window of at least 6 months to capture dispensed prescriptions of interest.

### Statistical methods

We used *n* (%) to describe categorical variables and means (s.d.) and medians (IQR) to describe continuous variables of the study population. We used time since index date as time scale in our time-to-event models.

To estimate the cumulative probability of developing AMI in patients with SSc and the comparators, we used the Kaplan–Meier method. We estimated the crude incidence rates of AMI in both groups and stratified by sex in addition to the crude rate differences. We used flexible parametric models to estimate the age- and sex-specific hazard ratios (HRs) of AMI. We also used flexible parametric models [16] adjusted for age (as a continuous variable), sex (women *vs*. men), education level (classified into ≤ 9 years, 10–12 years and > 12 years), comorbidities of diabetes mellitus (present *vs*. absent), hypertension (present *vs*. absent), dyslipidaemia (present *vs*. absent) and renal diseases (present *vs*. absent) and allowing for time-dependent effect of SSc, to study the relative risk of AMI over time since start of follow-up. We tested the remaining covariates using Aalen method and none of them had a time-varying effect. To compare AMI hazard over age in patients with SSc and their comparators, we estimated crude AMI hazard over age using flexible parametric model using increasing age as time scale.

As a sensitivity analysis to explore the risk of truly incident AMI unrelated to previously undetected ischaemic heart disease that may have been detected during the extensive screening process patients with SSc usually undergo when diagnosed leading to a hospitalization, we started follow-up 30 days after index date and estimated the absolute and relative risk of AMI thereafter. We conducted another sensitivity analysis where we ascertained the occurrence of AMI exclusively from hospitalizations with a discharge code indicating AMI. We considered participants who had AMI coded as the underlying or the contributing cause of death from the CDR censored. The rationale behind this sensitivity analysis is that data from hospitalizations is considered more reliable than data from the CDR where the cause of death is obtained from death certificates.

To study the clinical outcomes of AMI, we used a cohort study design comprising patients with SSc and matched comparators who developed AMI starting from 1 January 2006, ascertained only from hospitalizations. Start of follow-up was the date of discharge due to AMI. The first outcome was all-cause 30-day readmission, where we followed participants until the outcome, death, emigration or 30 days after discharge. In another analysis, the outcome was all-cause mortality where we followed participants until the outcome, emigration or study end. We used flexible parametric models to estimate HR of the outcome adjusted for age at AMI onset, sex, education level at AMI onset and the above-mentioned comorbidities at AMI onset.

We performed the statistical analyses using the packages ‘survival’ [17], ‘biostat3’ [18] and ‘rstpm2’ [19] in R version 4.3.2 (R foundation for statistical computing, Vienna, Austria) [20].

## Results

We identified 1722 patients with incident SSc and 16 983 matched comparators. Due to a history of ischaemic heart disease prior to index date, we excluded 143 (8%) of patients with SSc and 919 (5%) of the comparators. More than 20% of those patients with SSc received their first ever code for ischaemic heart disease within 1 year before index date compared with less than 10% of those from the comparators (Supplementary Fig. S1, available at *Rheumatology Advances in Practice* online).

The total study population included therefore 1579 patients with SSc and 16 064 general population comparators. The mean age at index date was 58 (s.d. 14.8) and 58 (s.d. 14.8) years in patients with SSc and the comparators, respectively. Women represented 82% of each of the two groups. The total follow-up time was 9581 years, median 5.2 (IQR 6.7) for the SSc cohort and 110, 724 years, median 6.3 years (IQR 7.3) for the comparators. Characteristics at index date are presented in Table 1.

**Table 1.** Characteristics at index date of patients with SSc diagnosed between 2004 and 2019 and their matched general population comparators with no history of ischaemic heart disease

	Patients with SSc	General population comparators
*N*	1579	16 064
Age at start of follow-up, years, mean (s.d.)	58 (14.8)	58 (14.8)
Sex		
Women, *n* (%)	1295 (82%)	13 098 (82%)
Men, *n* (%)	284 (18%)	2966 (18%)
Highest attained education, *n* (%)		
≤ 9 years	355 (23%)	3675 (23%)
10–12 years	668 (42%)	6769 (42%)
> 12 years	505 (32%)	5306 (33%)
Missing	51 (3%)	314 (2%)
Comorbidities at index date^a^, *n* (%)		
Diabetes	85 (6%)	955 (7%)
Hypertension	513 (37%)	3521 (25%)
Dyslipidaemia	196 (14%)	2165 (15%)
Renal diseases	66 (5%)	368 (3%)

a Comorbidities at index date were identified only in participants with index date starting from 1 January 2006 (1403 patients with SSc and 14 308 matched comparators) since data from the National Prescribed Drug Register are available starting from 1 July 2005.

During follow-up, 53 patients with SSc were hospitalized for incident AMI and 19 patients died due to incident AMI; in total, 72 patients with SSc were classified as having had AMI (4.6%). Of the comparators, 320 were hospitalized for incident AMI and 95 died due to incident AMI; in total, 415 (2.6%). Patients with SSc were younger (mean age 71 years, s.d. 10.1) than the comparators (mean age 75 years s.d. 9.5) upon having the outcome. The cumulative probability of incident AMI was significantly higher in patients with SSc than the matched comparators throughout follow-up (*P* < 0.0001). In patients with SSc, the 1-year cumulative probability of incident AMI was 1.0%, the 5-year probability was 3.3%, and the 10-year probability was 7.7%, compared with 0.3%, 1.6% and 3.5%, respectively, in the comparators. The crude incidence rate of AMI was 75.2 (95% CI 58.8–94.6) per 10 000 person-years in patients with SSc and 37.5 (95% CI 34.0–41.3) per 10 000 person-years in the comparators. Patients with SSc had 140% higher risk of AMI compared with the comparators; age- and sex-adjusted HR 2.4 (95% CI 1.9–3.0). Similarly, women and men from the SSc group had higher incidence rates of AMI than their counterparts from the comparators (61.2 *vs*. 29.5 and 138.6 *vs*. 71.0 per 10 000 person-years, respectively) with statistically significant rate differences and HRs (Table 2). The age- and sex-adjusted HR was 2.5 (95% CI 1.8–3.2) in women and 2.2 (95% CI 1.4–3.2) in men.

**Table 2.** Incidence rate of acute myocardial infarction (AMI) in patients with SSc and general population comparators, and stratified by sex, in addition to crude rate differences and hazard ratios (HRs)

	Patients with SSc (*n* = 1579)			General population comparators (*n* = 16 064)				
	*n*	Person-years	Crude IR with 95% CI	*n*	Person-years	Crude IR with 95% CI	Crude rate difference with 95% CI	Adjusted HR with 95% CI^a^
All individuals	72	9581	75.2 (58.8–94.6)	415	110 724	37.5 (34.0–41.3)	37.7 (19.9–55.4)	2.4 (1.9–3.0)
Women	48	7850	61.2 (45.1–81.1)	264	89 463	29.5 (26.1–33.3)	31.6 (14.0–49.3)	2.5 (1.8–3.2)
Men	24	1731	138.6 (88.8–206.3)	151	21 261	71.0 (60.1–83.3)	67.6 (11.0–124.2)	2.2 (1.4–3.2)

IR incidence rate and rate difference are per 10 000 person-years.

a Age- and sex-adjusted HR in the entire cohort. Age-adjusted in women and men, respectively.

**Figure 1.**
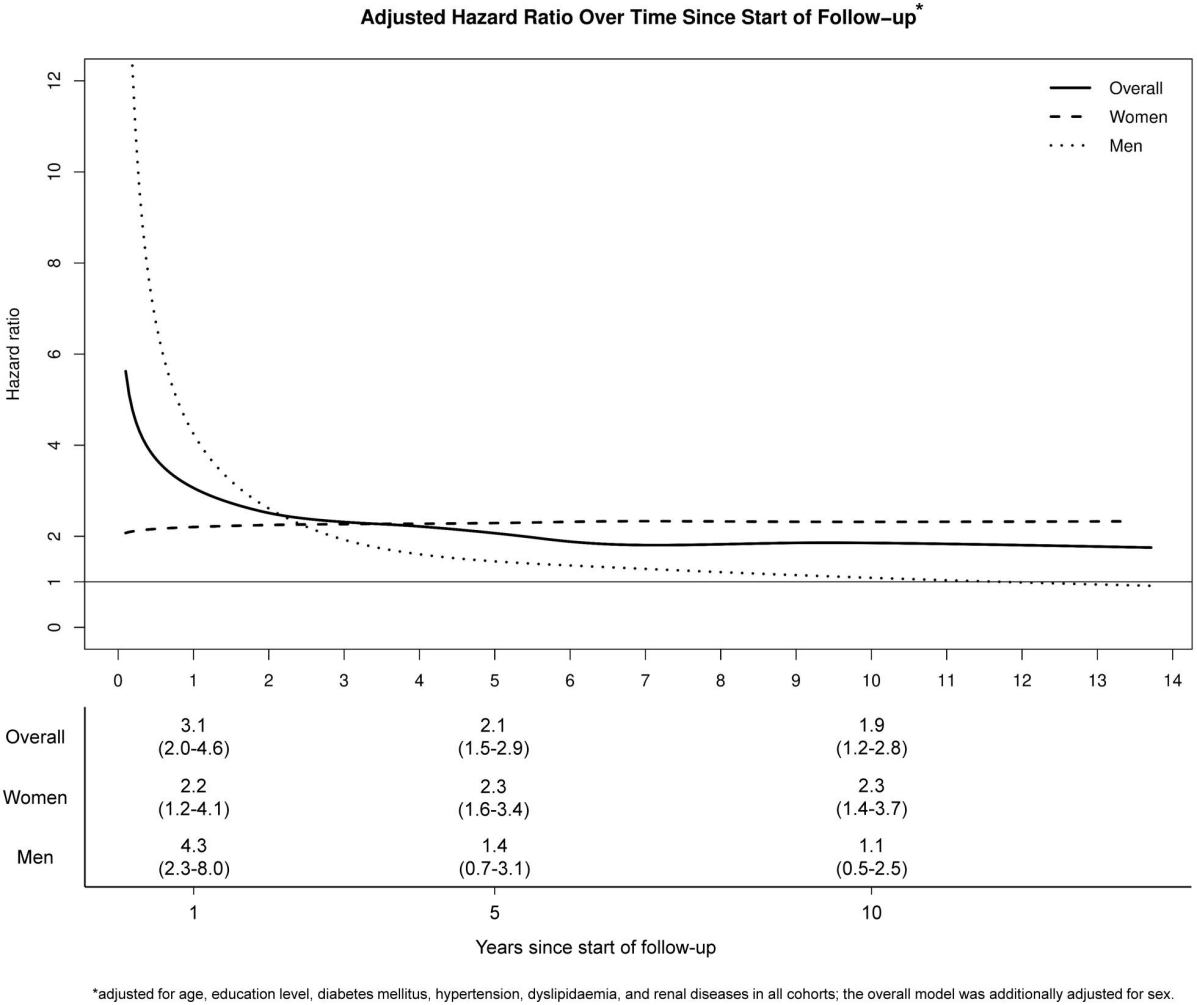
Hazard ratio of acute myocardial infarction (AMI) in patients with SSc compared with the general population comparators with index date starting from 1 January 2006 (1403 patients with SSc and 14 308 matched comparators), overall and stratified by sex, using flexible parametric models and allowing for time-dependent effect of SSc, adjusted for age, education level, diabetes mellitus, hypertension, dyslipidaemia and renal diseases in all cohorts, the overall model was additionally adjusted for sex

After adjusting for age, sex, education level and traditional risk factors for AMI (diabetes mellitus, hypertension, dyslipidaemia and renal disease) and by allowing for time-dependent effect of SSc, patients with SSc had a higher rate of AMI throughout the entire follow-up period (Fig. 1). This relative risk was highest at the end of the first year since start of follow-up, HR 3.1 (95% CI 2.0–4.6) and then declined to 2.1 (95% CI 1.5–2.9) at the end of the fifth year and to 1.9 (95% CI 1.2–2.8) at the end of the 10th year since start of follow-up. Similarly, HR in men was highest in the beginning of follow-up period and then declined, while in women, it was rather stable throughout follow-up period (Fig. 1). HRs for AMI overall, men and women with CI are shown in Supplementary Fig. S2, available at *Rheumatology Advances in Practice* online.

**Figure 2.**
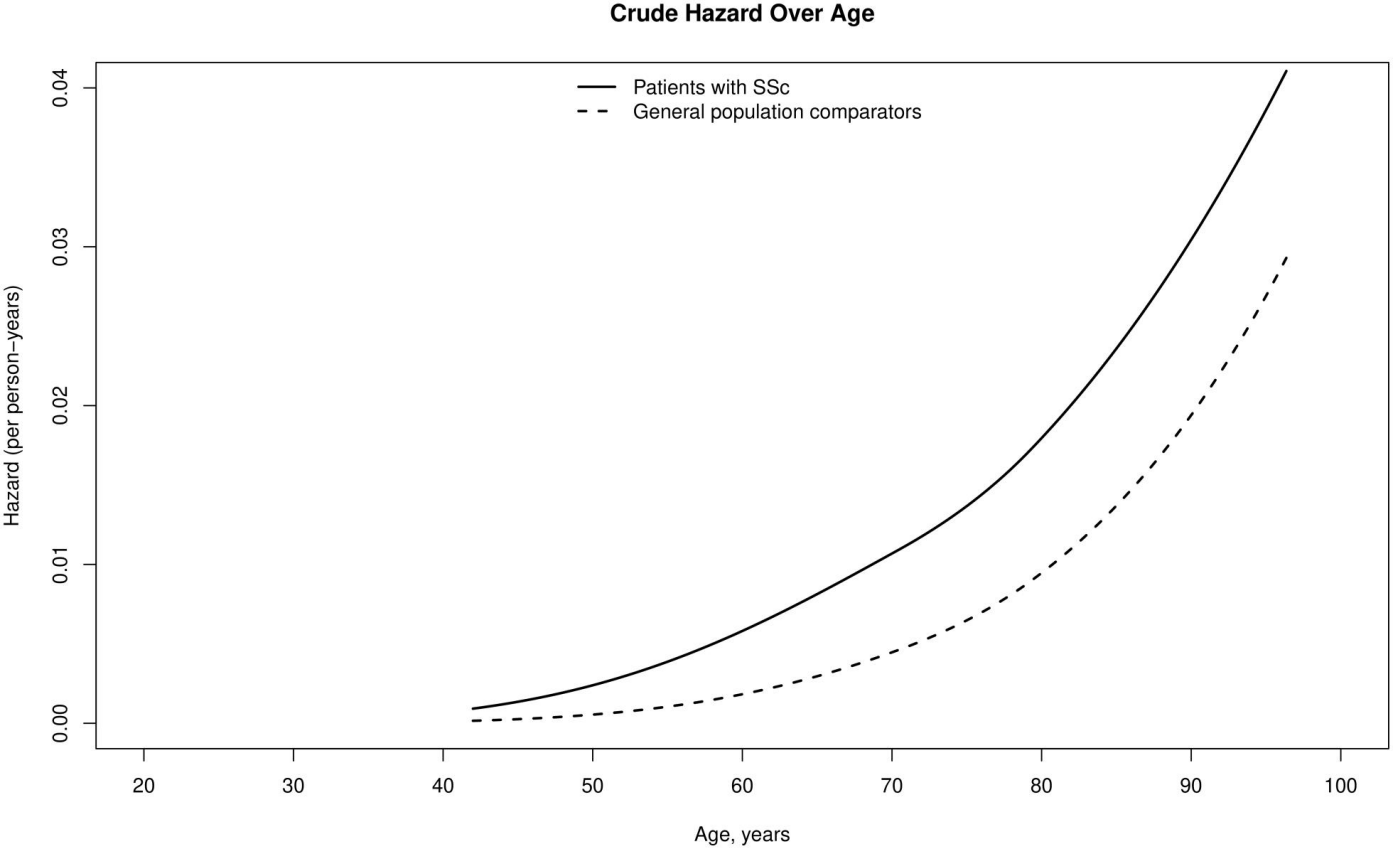
Crude hazard of acute myocardial infarction (AMI) in patients with SSc and the general population comparators using flexible parametric model and using age as time scale

In a model using increasing age as time scale, AMI hazard in patients with SSc increased more rapidly with increasing age compared with the general population comparators (Fig. 2). In both patients with SSc and the matched comparators, the first AMI case occurred after the age of 40 years.

In the first sensitivity analysis, where we started follow-up 30 days after index date, the results were in line with the main findings. The cumulative probability of incident AMI was still significantly higher in patients with SSc than the comparators throughout follow-up (*P* < 0.0001); the 1-year cumulative probability was 0.6% *vs*. 0.2%, the 5-year probability was 2.2% *vs*. 1.3%, and the 10-year probability was 6.1% *vs*. 2.9% in patients with SSc and the comparators, respectively. Similarly, AMI incidence rates were higher in the SSc group (Supplementary Table S1, available at *Rheumatology Advances in Practice* online). The fully adjusted HR with start of follow-up from 1 January 2006 was still highest in the first year after start of follow-up and then decreased gradually. The pattern of HRs was however rather fluctuating in women and men, most likely due to fewer AMI cases identified in each stratum (Supplementary Fig. S3, available at *Rheumatology Advances in Practice* online). The results of the second sensitivity analysis where we ascertained the occurrence of AMI exclusively from hospitalizations did not alter our interpretation from the main findings (Supplementary Table S2 and Supplementary Fig. S4, available at *Rheumatology Advances in Practice* online).

There were 50 patients with SSc and 314 comparators who were hospitalized due to incident AMI starting from 1 January 2006. Mean age at AMI onset was 69.7 years for patients with SSc and 74.0 years for the comparators (Table 3). Of patients with SSc, 18 (36%) were re-admitted within 30 days of discharge compared with 98 (31%) of the comparators corresponding to an adjusted HR of 1.3 (95% CI 0.7–2.0). During follow-up, 19 (38%) patients with SSc and 93 (30%) of the comparators died. Patients with SSc were at significantly higher risk of all-cause mortality, adjusted HR of 2.7 (95% CI 1.6–4.4).

**Table 3.** Characteristics at date of acute myocardial infarction (AMI) onset of patients with SSc and their matched general population comparators between 2006 and 2019

	Patients with SSc	General population comparators
*N*	50	314
Age at AMI date, years, mean (s.d.)	69.7 (11.1)	74.0 (9.3)
Sex		
Women, *n* (%)	32 (64%)	191 (61%)
Men, *n* (%)	18 (36%)	123 (39%)
Highest attained education, *n* (%)		
≤ 9 years	11 (22%)	121 (39%)
10–12 years	23 (46%)	121 (39%)
> 12 years	12 (24%)	50 (15%)
Missing	4 (8%)	22 (7%)
Comorbidities at AMI date, *n* (%)		
Diabetes	5 (10%)	65 (21%)
Hypertension	34 (68%)	183 (58%)
Dyslipidaemia	15 (30%)	119 (38%)
Renal diseases	7 (14%)	25 (8%)

## Discussion

This nationwide population-based study demonstrated that patients with SSc in Sweden without a prior history of ischaemic heart disease are at increased risk for developing AMI compared with matched comparators from the general population. As observed in previous reports, this association is independent of traditional risk factors for AMI.

The pathophysiology of AMI in SSc is complex and not fully understood. The role of atherosclerosis of coronary arteries and microvasculature impairment have both been suggested. A study on all AMI hospitalizations in a large healthcare database in the USA reported that patients with SSc were not more likely to undergo invasive management compared with controls without autoimmune diseases [21]. Previous case series reported that patients with SSc are more likely to have normal coronary arteries than controls when admitted due to AMI [22]. Likewise, a similar prevalence of coronary artery disease evaluated using coronary angiography was shown in patient with SSc and SSc-free individuals [23]. On the other hand, coronary calcium score as a marker for coronary atherosclerosis was found to be higher in asymptomatic patients with SSc than matched healthy individuals [24, 25].

In our present study, we aimed to study the risk of AMI in a cohort of patients with SSc and comparators with no previous history of ischaemic heart disease, such as stable and unstable angina. We estimated the crude incidence rate of AMI in patients with SSc to be 75.2 per 10 000, compared with 37.5 in the comparators. Patients with SSc were at significantly increased risk of AMI compared with the comparators. This association remained significant even after adjusting for age, sex, education level as a proxy for socioeconomic status and traditional risk factors for AMI: diabetes, hypertension, dyslipidaemia and renal disease, in accordance with previous studies [6–9, 26]. A population-based study in Taiwan reported a 2.5 increased risk (HR: 2.5, 95% CI 1.6–3.8) of AMI in patients with SSc comparted to matched comparators after adjusting for traditional risk factors for AMI [6]. Similarly, a study in the USA reported an adjusted HR of 4.9 (95% CI 1.2–19.7) [7]. A Canadian study found that patients with SSc had over 3-fold increased risk of AMI, adjusted HR 3.5 (95% CI 2.5–4.8) [8]. In Denmark, adjusted HR for AMI in patients with SSc was 1.9 (95% CI 1.5–2.5) [9]. Our findings support therefore the role of SSc-related mechanisms in the development of AMI. Another finding is that more than 20% of patients with SSc who were excluded due to ischaemic heart disease received their first ever code for ischaemic heart disease within 1 year before index date. Since diagnostic delay usually occurs in SSc, those cases might be associated with SSc and our estimates might be underestimating the true rate of AMI. On the contrary, less than 10% of the comparators received their first ever code for ischaemic heart disease within 1 year before index date.

We found the risk of AMI to be highest after SSc diagnosis and then it decreased slowly during follow-up (Fig. 1). We observed this pattern also in the sensitivity analysis when we started follow-up 30 days after index date indicating that the increased risk of AMI short after SSc diagnosis is more likely to be related to SSc-specific mechanisms, rather than extensive screening. A possible explanation to this finding is high disease activity at diagnosis before accomplishing a plausible control of the disease using SSc-targeting drugs. Similar observation was reported by Aviña-Zubieta et al. who reported the risk of myocardial infarction to be highest in the first year following the diagnosis of SSc and then it declined gradually [27]. Stratified by sex, we found this pattern more prominent in men in our cohort, while the risk of AMI was rather stable over follow-up period in women (Fig. 1). In a report from the EULAR scleroderma trials and research group (EUSTAR) on gender differences in patients with very early SSc (less than 1 year since the onset of non-Raynaud symptom) and patients with early SSc (less than 3 years since the onset of non-Raynaud symptom) [28], men were found to have higher disease activity than women, defined using the SSc activity score proposed by the European Scleroderma Study Group [29]. Furthermore, elevated acute phase reactants were more prevalent in men than in women [28]. This issue might explain the observed high risk of AMI short after SSc diagnosis in men, but not in women. The relatively low number of men with SSc in the cohort should be taken into consideration here since depletion of susceptibles might partially contribute to this pattern of AMI risk over time.

Increasing age is an independent risk factor for cardiovascular diseases, including myocardial infarction [30, 31]. Age at SSc diagnosis was found a predictor of AMI, with higher age being associated with higher AMI risk in a population-based study in Taiwan [6]. However, a Danish study reported that the risk of AMI in patients with SSc aged ≥ 55 years at diagnosis was not higher than the background population while the risk was significantly higher in those < 55 years at SSc diagnosis [32]. In our study, we used age as time scale to explore how the risk of AMI changes over increasing age in patients with SSc, rather than studying the AMI risk depending on age at SSc diagnosis. We found the AMI risk increasing more rapidly over increasing age in patients with SSc compared with their matched comparators (Fig. 2).

Previous research showed that AMI in patients with SSc is associated with worse outcome and significantly higher risk of mortality compared with individuals without autoimmune rheumatic diseases, adjusted odds ratio was 1.8 (95% CI 1.6–2.0) [21]. In agreement with that, we found that patients with SSc who developed AMI were at 170% higher risk of mortality (2.7 HR, 95% CI 1.6–4.4) than their matched comparators who developed AMI. Also, higher proportion of patients with SSc than the comparators were readmitted within 30 days of discharge; however, this difference was not statistically significant.

A major strength of this study is its design utilizing the well-established nationwide Swedish registers to comprise a large unselected cohort including all patients with incident SSc in Sweden in addition to matched comparators from the general population with the ability for long-term follow-up. The NPR’s complete coverage of all hospitalizations in Sweden in addition to the longstanding history of reporting causes of death using death certificates provided a reliable and unbiased source to ascertain our exposure (incident SSc diagnosis) and our outcome (incident AMI or death from incident AMI). However, this study has some limitations. The generalizability of our results to other populations can be limited due to the fairly unique structure of the Swedish healthcare system with equal access to all residents in addition to the fact that the Swedish population is composed mainly of Caucasians. We have no data on the coronary angiography pattern and management in patients with SSc and the comparators to explore whether the higher AMI risk in patients with SSc is primarily driven by coronary artery obstruction (type 1 AMI) or imbalance between oxygen demand and supply in the myocardium (type 2 AMI) caused possibly by microvascular dysfunction, pulmonary arterial hypertension or myocardial fibrosis. Type 2 AMI represents approximately 12% of all AMI types in the general population [33]. In patients with SSc, type 2 AMI is probably more likely to be present since they are prone to be hospitalized due to other conditions where elevated levels of cardiac enzymes can be detected leading to ICD code indicating AMI. Second, we did not investigate the potential difference in AMI management between patients with SSc and the comparators, including percutaneous coronary intervention (PCI) and coronary artery bypass graft (CABG). In addition, the lack of data on other traditional risk factors for AMI, such as physical activity, smoking status, and BMI, is a limitation. SSc-specific characteristics like autoantibody pattern and SSc disease subset are not captured in the used registers, and we were therefore unable to stratify the risk of AMI according to these parameters. To the best of our knowledge, the role of these characteristics in the risk of developing AMI has not been studied yet in a population-based cohort. Further studies are therefore warranted.

In conclusion, this study, in line with previous reports, suggests that SSc is associated with higher risk of incident AMI, even in patients with no history of ischaemic heart disease. The risk of AMI increases more rapidly over increasing age in patients with SSc compared with the general population. Our findings encourage close surveillance of patients with SSc, especially at diagnosis. The 2-fold increased risk of AMI in SSc also stress the importance of identifying and treating modifiable risk factors for AMI.

## Supplementary material

Supplementary material is available at *Rheumatology Advances in Practice* online.

## Supplementary Material

rkaf054_Supplementary_Data

## Data Availability

Data are available upon reasonable request.
