# Supplementary material for: Increased risk of acute myocardial infarction in Swedish patients with systemic sclerosis: a population-based study
Source: Rheumatol Adv Pract. 2025 May 9;9(2):rkaf054. doi: 10.1093/rap/rkaf054 (PMC12106857; doi:10.1093/rap/rkaf054)
Supplement: rkaf054_Supplementary_Data [file rkaf054_supplementary_data.zip › Supplementary file.pdf]

Increased risk of acute myocardial infarction in Swedish patients with systemic sclerosis: a population-based study

Majd Bairkdar[1], Karina Patasova[1], Pontus Andell[2, 3], Marie Holmqvist[1, 4]

[1]Clinical epidemiology division, Department of medicine Solna, Karolinska Institutet.

[2]Department of Physiology and Pharmacology, Karolinska Institutet, Stockholm, Sweden

[3]Medical Unit Cardiology, Karolinska University Hospital, Stockholm, Sweden

[4]Medical Unit Gastroenterology, Dermatolgy and Rheumatology, Karolinska University Hospital, Stockholm, Sweden

Supplementary file

Supplementary data 1. Comorbidities definition at index date using the National Patient Register and the National Prescribed Drug Register.

Diabetes mellitus:

either any visit prior to index date (ICD-10: E10-E14) as a main or secondary diagnosis or at least two dispensed prescriptions of any glucose-lowering drug (ATC: A10) prior to index date. This definition is considered as a good proxy for pharmacologically treated type 1 and 2 diabetes in Sweden (1).

Hypertension:

either any visit prior to index date (ICD-10: I10-I15) as a main or secondary diagnosis or at least one dispensed prescription of at least two of the following classes of antihypertensive agents prior to index date (2): α adrenergic blockers (ATC: C02A, C02B, C02C), non-loop diuretics (ATC: C02DA, C02L, C03A, C03B, C03D, C03E, C03X, C07C, C07D, C08G, C09BA, C09DA, C09XA52), vasodilators (ATC: C02DB, C02DD, C02DG, C04, C05), β blockers (ATC: C07), calcium channel blockers (ATC: C07F, C08, C09BB, C09DB), and renin-angiotensin system inhibitors (ATC: C09).

Dyslipidaemia:

either any visit prior to index date (ICD-10: E78) as a main or secondary diagnosis or at least two dispensed prescriptions of lipid modifying agents (ATC: C10) prior to index date (2).

Renal disease:

any visit prior to index date (ICD-10: N00– N19) as a main or contributory diagnosis.

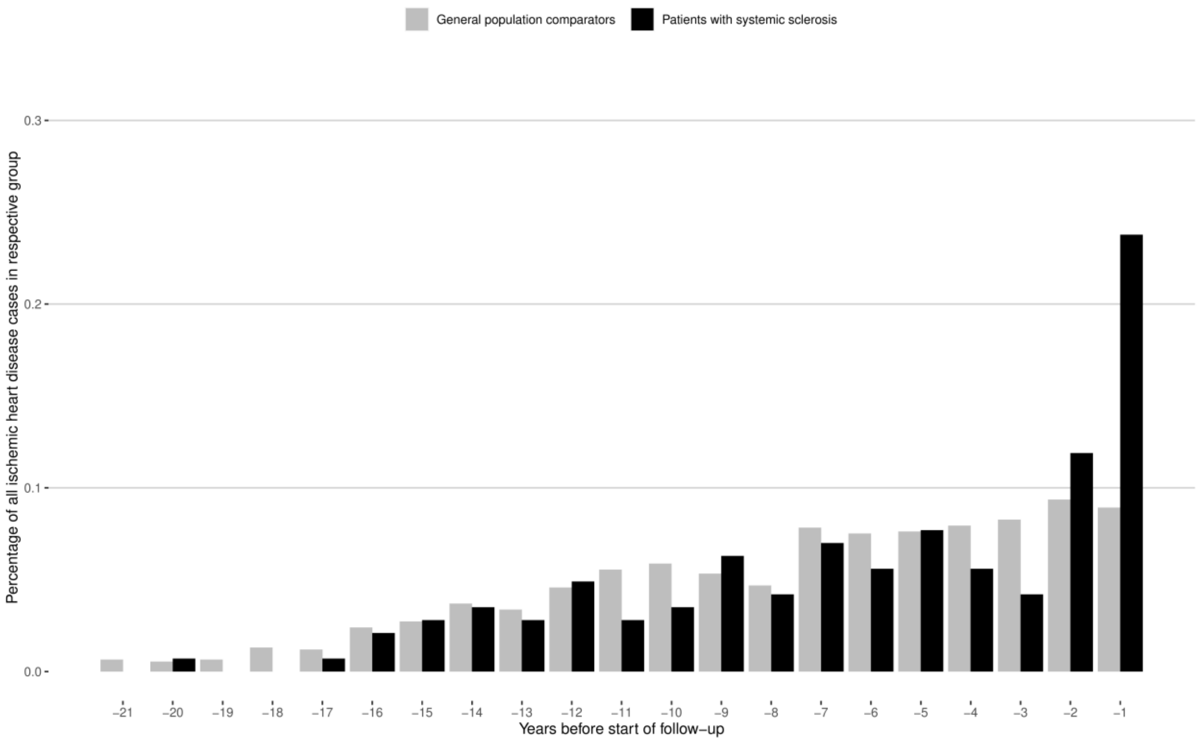

Supplementary figure 1. Percentage of all ischemic heart disease cases in patients with systemic sclerosis and their comparators, respectively, over years prior to start of follow-up.

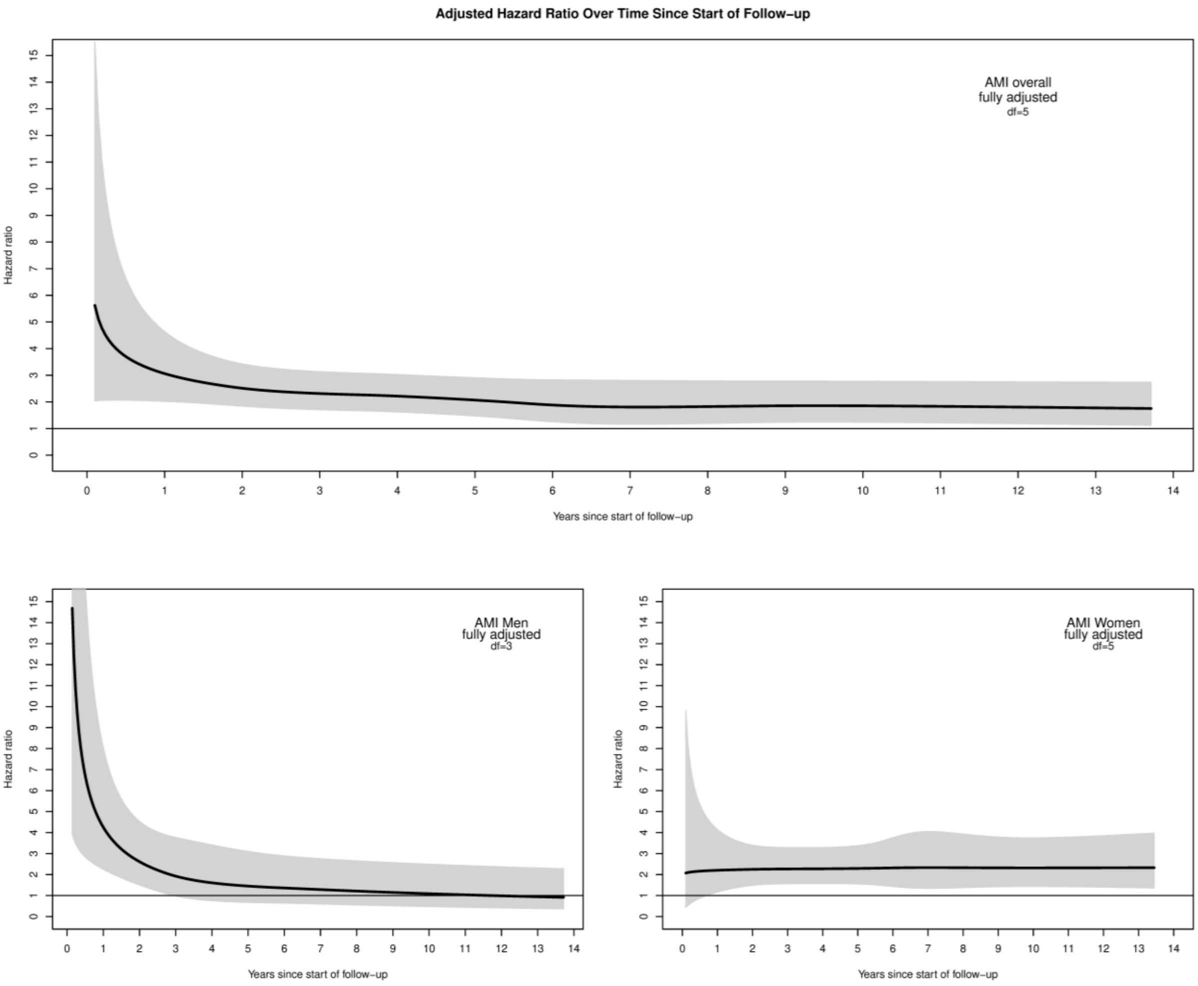

Supplementary figure 2. Hazard ratio of acute myocardial infarction (AMI) in patients with SSc compared to the general population comparators with **index date starting from 1 January 2006 (1403 patients with SSc and 14308 matched comparators) with confidence intervals**, overall and stratified by sex, using flexible parametric models adjusted for age, sex, education level, diabetes mellitus, hypertension, dyslipidaemia, and renal diseases and allowing for time-dependent effect of SSc.

Sensitivity analysis 1

To explore the risk of truly incident AMI unrelated to previously undetected ischemic heart disease that may have been detected during the extensive screening process patients with SSc usually undergo when diagnosed leading to a hospitalization, we started follow up 30 days after index date and estimated the absolute and relative risk of AMI thereafter. The results are shown below.

Supplementary table 1. Incidence rate of acute myocardial infarction (AMI) in patients with SSc and general population comparators with start of follow-up 30 days after index date, stratified by sex, in addition to crude rate differences and hazard ratios (HRs).

	**Patients with SSc (n=1 547)**			**General population comparators (n=15 908)**				
	n	Person-years	Crude IR with 95% CI	n	Person-years	Crude IR with 95% CI	Crude rate difference with 95% CI	Adjusted HR with 95% CI*
**All individuals**	52	9 378	55.4 (41.4-72.7)	319	108 888	29.3 (26.2-32.7)	26.2 (10.7-41.6)	2.2 (1.7-2.9)
**Women**	33	7 683	43.0 (29.6-60.3)	191	87 965	21.7 (18.7-25.0)	21.2 (6.3-36.2)	2.4 (1.6-3.2)
**Men**	19	1 695	112.1 (67.5-175.0)	128	20 923	61.2 (51.0-72.7)	50.9 (-0.6-102.4)	2.1 (1.3-3.1)

IR *incidence rate and rate difference are per 10,000 person-years.*
**age- and sex-adjusted HR in the entire cohort. Age-adjusted in women and men, respectively.*

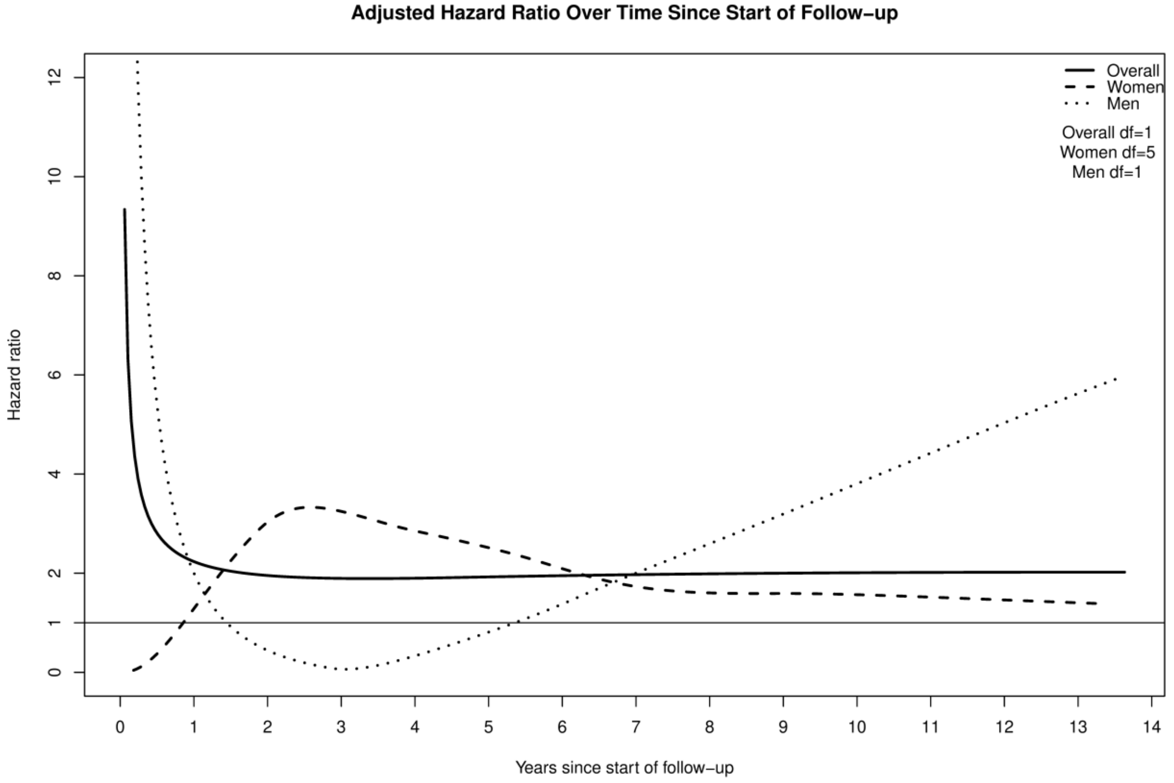

Supplementary figure 3. Hazard ratio of acute myocardial infarction (AMI) in patients with SSc compared to the general population comparators with **index date starting from 1 January 2006 (1376 patients with SSc and 14173 matched comparators) with start of follow-up 30 days after index date**, overall and stratified by sex, using flexible parametric models adjusted for age, sex, education level, diabetes mellitus, hypertension, dyslipidaemia, and renal diseases and allowing for time-dependent effect of SSc.

Sensitivity analysis 2

To explore the risk of truly incident acute myocardial infarction (AMI) ascertained exclusively from hospitalizations with a discharge code indicating AMI. Participants who had AMI coded as the underlying or the contributing cause of death from the Cause of Death Register (CDR) will be considered censored. The rationale behind this sensitivity analysis is that data from hospitalizations is considered more reliable than data from the CDR where the cause of death is ascertained from death certificates. The results are shown below.

Supplementary table 2. Incidence rate of acute myocardial infarction (AMI) ascertained exclusively from hospitalizations in patients with SSc and general population comparators, stratified by sex, in addition to crude rate differences and hazard ratios (HRs).

	**Patients with SSc (n=1 547)**			**General population comparators (n=15 908)**				
	n	Person-years	Crude IR with 95% CI	n	Person-years	Crude IR with 95% CI	Crude rate difference with 95% CI	Adjusted HR with 95% CI*
**All individuals**	53	9 581	55.3 (41.4-72.4)	320	110724	28.9 (25.8-32.2)	26.4 (11.2-41.6)	2.3 (1.7-2.9)
**Women**	34	7 850	43.3 (30.0-60.5)	192	89 463	21.5 (18.5-24.7)	21.9 (7.0-36.7)	2.4 (1.7-3.3)
**Men**	19	1 731	109.8 (66.1-171.4)	128	21 261	60.2 (50.2-71.6)	49.5 (-0.9-100.0)	2.0 (1.3-3.1)

IR *incidence rate and rate difference are per 10,000 person-years.*
**age- and sex-adjusted HR in the entire cohort. Age-adjusted in women and men, respectively.*

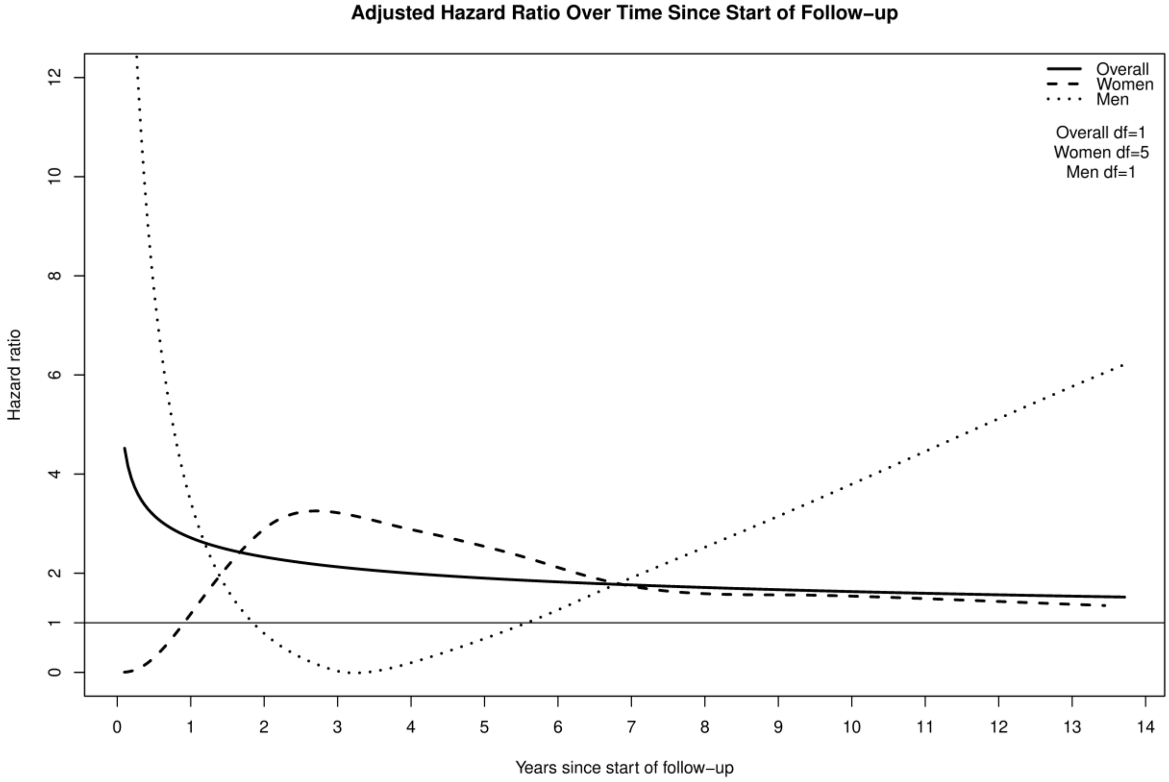

Supplementary figure 4. Hazard ratio of acute myocardial infarction (AMI) ascertained exclusively from hospitalizations in patients with SSc compared to the general population comparators with **index date starting from 1 January 2006 (1403 patients with SSc and 14308 matched comparators)**, overall and stratified by sex, using flexible parametric models adjusted for age, sex, education level, diabetes mellitus, hypertension, dyslipidaemia, and renal diseases and allowing for time-dependent effect of SSc.

References

1. Andersson T, Ahlbom A, Carlsson S. Diabetes Prevalence in Sweden at Present and Projections for Year 2050. PLoS One. 2015;10(11):e0143084.
2. Hvidberg MF, Johnsen SP, Glumer C, Petersen KD, Olesen AV, Ehlers L. Catalog of 199 register-based definitions of chronic conditions. Scand J Public Health. 2016;44(5):462-79.
